# Nuclear receptor 4A1 Regulates Mitochondrial Homeostasis in Cardiac Post-Ischemic Injury by Controlling Mitochondrial Fission 1 Protein-Mediated Fragmentation and Parkin-Dependent Mitophagy

**DOI:** 10.7150/ijbs.104680

**Published:** 2025-01-01

**Authors:** Haoran Ye, Jialong Lin, Hui Zhang, Jing Wang, Yuan Fu, Zhaopei Zeng, Junmeng Zheng, Jun Tao, Junxiong Qiu

**Affiliations:** 1 Department of Cardiovascular Surgery, Sun Yat-sen Memorial Hospital, Sun Yat-sen University, Guangzhou, China 510120.; 2 Department of Cardiovascular Medicine, The Fourth Affiliated Hospital of Guangzhou Medical University, Zengcheng District People's Hospital of Guangzhou, Guangzhou, China, 511300.; 3 Guang'anmen Hospital China Academy of Chinese Medical Sciences, China.; 4 Xianning Medical College, Hubei University of Science & Technology, Xianning 437000, China.

**Keywords:** Cardiac post-ischemic damage, Nr4a1, Fis1, Parkin, Mitochondrial fission and Mitophagy

## Abstract

The close interaction of mitochondrial fission and mitophagy, two crucial mechanisms, is key in the progression of myocardial ischemia-reperfusion (IR) injury. However, the upstream regulatory mechanisms governing these processes remain poorly understood. Here, we demonstrate a marked elevation in Nr4a1 expression following myocardial IR injury, which is associated with impaired cardiac function, heightened cardiomyocyte apoptosis, exacerbated inflammatory responses, and endothelial dysfunction. Notably, Nr4a1-knockout mice exhibited remarkable resistance to acute myocardial IR injury, characterized by preserved mitochondrial integrity relative to their wild-type counterparts. Functional analyses revealed that elevated Nr4a1 expression after IR injury promotes Fis1-mediated mitochondrial fission while suppressing Parkin-driven mitophagy. Importantly, interventions that inhibit mitochondrial fission or enhance mitophagy effectively ameliorated IR-induced cardiomyocyte and endothelial dysfunction. Collectively, these results highlight that the absence of Nr4a1 provides a shield against cardiac post-ischemic damage by reinstating balance within the mitochondria through inhibiting Fis1-induced fission and promoting Parkin-triggered mitophagy. Furthermore, therapeutic strategies targeting the Nr4a1/mitochondria axis may offer promising avenues for improving cardiac outcomes under myocardial IR stress.

## Introduction

In the realm of global health challenges, acute myocardial infarction stands as a prominent contributor to illness and death, emphasizing the urgency of swift interventions like percutaneous coronary intervention (PCI) to reestablish blood circulation in blocked coronary arteries. Paradoxically, while these interventions are critical for tissue salvage, they frequently induce ischemia-reperfusion (IR) injury, exacerbating myocardial damage 1. Clinical data indicate that myocardial IR injury affects approximately 10-50% of patients undergoing reperfusion therapy 2, 3, with a persistently high 30-day mortality rate 4. This alarming statistic underscores the need to unravel the molecular underpinnings of IR injury to inform the development of more effective therapeutic approaches.

Recent studies have increasingly highlighted the pivotal influence of mitochondrial dysfunction in the pathophysiology of post-ischemic myocardial injury. Both mitochondrial fission 5, 6 and mitophagy are recognized as critical determinants of cellular fate during IR stress 7-9. Excessive mitochondrial fission leads to pathological fragmentation, disrupting energy production and precipitating apoptosis 10, 11. Conversely, mitophagy—a selective autophagic process targeting damaged mitochondria—plays a protective role by maintaining mitochondrial integrity and cellular viability 12. Despite the recognized importance of these processes, the regulatory networks governing the interplay between mitochondrial fission and mitophagy remain incompletely defined.

Fresh insights indicate that nuclear receptor subfamily 4 group A member 1 (Nr4a1) plays a pivotal role in regulating mitochondrial dynamics. A recent investigation demonstrated that Nr4a1 intensifies hepatic damage by enhancing Drp1-driven mitochondrial fission while inhibiting Bnip3-mediated mitochondrial autophagy 13. Likewise, Nr4a1 has been linked to the advancement of heart failure 14 and oxidative stress-induced cardiomyocyte apoptosis 15. Together, these discoveries imply that Nr4a1 could play a role in the pathological aspects of post-ischemic myocardial damage by disturbing the balance within mitochondria.

Within the realm of IR injury, Drp1 primarily governs the process of mitochondrial fission, a cytoplasmic protein that translocates to the mitochondrial outer membrane via receptors such as mitochondrial fission 1 protein (Fis1) 6, 10. Once recruited, Drp1 assembles into constriction rings that fragment mitochondria into smaller units 16. Concurrently, mitophagy ensures the clearance of dysfunctional mitochondria, maintaining mitochondrial quality and preventing excessive damage 17, 18. Key mediators of mitophagy, including Parkin, FUN14 domain-containing 1 (FUNDC1), and Bnip3, facilitate the autophagic flux necessary for mitochondrial turnover 5, 8, 10. However, the precise contribution of these pathways to microvascular IR injury, particularly in the context of Nr4a1 regulation, remains poorly understood.

This study aims to elucidate the mechanistic role of Nr4a1 in affecting Fis1-mediated mitochondrial fission and Parkin-associated mitophagy during cardiac microvascular IR injury. By investigating the dual regulatory effects of Nr4a1 on these processes, we seek to uncover novel therapeutic targets for mitigating the deleterious effects of IR injury and improving clinical outcomes.

## Materials and Methods

### *In vivo* model of myocardial ischemia-reperfusion (IR) injury

Animal experiments were conducted following the guidelines set by the Guide for the Care and Use of Laboratory Animals and approved by the Institutional Animal Care and Use Committees. Twelve-week-old male NR4A1 knockout (NR4A1-KO) mice and their wild-type (WT) counterparts were employed in the study. The ischemia-reperfusion (IR) model was induced by temporarily ligating the left anterior descending (LAD) coronary artery with an 8-0 silk suture. Ischemia lasted for 45 minutes, followed by variable periods of reperfusion (ranging from immediate to 24 hours). Myocardial pallor confirmed LAD occlusion, while tissue color restoration indicated reperfusion. Serum samples were collected post-reperfusion to analyze cardiac injury markers, such as creatine kinase-MB (CK-MB), troponin T, and lactate dehydrogenase (LDH), using ELISA kits from R&D Systems 19.

### Hypoxia reoxygenation injury (HR injury) model *in vitro*

Cardiac microvascular endothelial cells (CMECs) and cardiomyocytes were isolated from WT and NR4A1-KO mice. To simulate ischemia-reperfusion injury, cells were subjected to hypoxia-reoxygenation (HR) 20. Hypoxia was induced in a tri-gas incubator with 95% nitrogen and 5% carbon dioxide for 45 minutes, followed by reoxygenation under normoxic conditions for six hours. This model closely mimics the physiological conditions of ischemia-reperfusion injury, allowing for controlled assessments of mitochondrial function, oxidative stress, and apoptotic pathways 21.

### Western blot

Cellular extracts were obtained through a specific lysis protocol involving a tailored buffer solution that included enzyme inhibitors to prevent protein degradation. Following centrifugation at high speed under chilled conditions, the protein content was measured using a colorimetric method based on copper ion reduction. Standardized protein samples were then resolved by gel electrophoresis and transferred to a synthetic membrane for further analysis 22. Nonspecific binding sites on the membrane were saturated with a blocking agent derived from animal serum, after which the membrane was probed with a panel of primary antibodies directed against various cellular proteins, including nuclear receptors, mitochondrial regulators, and apoptosis-related factors 23. The membrane was subsequently treated with enzyme-linked secondary antibodies and exposed to a luminescent substrate to reveal the target protein bands. The resulting signal intensities were analyzed and quantified using specialized image processing software.

### Mitochondrial function and morphology

The integrity of the mitochondrial membrane was assessed using a fluorescent probe that accumulates in the mitochondrial matrix. After a brief incubation period at physiological temperature, the cells were examined under a specialized microscope to detect the probe's emission signals. The resulting fluorescence patterns were quantified by calculating the ratio of high-intensity aggregates to low-intensity monomers 24. To investigate mitochondrial structure, cells were labeled with antibodies targeting a specific outer membrane protein, and high-resolution images were acquired using a microscope equipped with a laser illumination system. Cellular energy status was evaluated using a bioluminescent assay that measures the concentration of a key energy metabolite. Additionally, the stability of the mitochondrial membrane was monitored by tracking changes in the fluorescence intensity of a potentiometric dye over time.

### Assessment of apoptosis and ROS production

Programmed cell death was evaluated through enzymatic labeling of DNA fragments and assays measuring the activity of key executioner enzymes 25. The proportion of cells undergoing programmed death was calculated relative to the total cell count. Oxidative stress was assessed by detecting fluorescent signals from a probe that reacts with free radicals, and the resulting fluorescence was quantified using a cell sorting technique 26. To specifically examine oxidative stress within mitochondria, a targeted fluorescent dye was employed. The accuracy of these findings was confirmed using pharmacological modulators that either suppress or enhance mitochondrial oxidative stress.

### Immunofluorescence and immunohistochemistry

Specimens were preserved in a fixative solution, then treated with a mild detergent to enhance permeability, and finally incubated in a blocking agent to minimize nonspecific binding. The samples were then probed with specific antibodies targeting various cellular proteins, including an endothelial enzyme, a nuclear receptor, and mitochondrial regulators, followed by staining with fluorescently labeled secondary antibodies 27. The resulting fluorescence patterns were visualized and recorded using a high-resolution microscope. For tissue sections, a staining protocol involving enzyme-linked antibodies was used, and the resulting signal intensity was measured and quantified 28.

### Evaluation of endothelial permeability and barrier function

Vascular barrier function was evaluated by measuring the passage of a fluorescent tracer and electrical conductivity across the endothelial layer 29. A monolayer of brain endothelial cells was subjected to hypoxic stress, and the subsequent changes in barrier permeability were assessed. The movement of the fluorescent tracer from the upper to lower compartment was quantified after a short incubation period. Electrical conductivity was also measured to assess the integrity of intercellular junctions 30.

### Gene silencing via siRNA

Cells were genetically modified to suppress the expression of specific mitochondrial regulators using a targeted RNA interference approach 31. After a prolonged incubation period, the effectiveness of the gene silencing was verified through protein analysis. The consequences of suppressing a key mitochondrial quality control protein on cellular processes were then investigated using functional assays 32.

### Statistical analysis

Results are expressed as averages with standard error margins. Statistical comparisons were made using a multi-group analysis of variance, with pairwise comparisons conducted using a specialized post-test. A threshold of 5% probability was used to determine significance. Computational analyses were carried out using a commercial data analysis platform.

## Results

### Nr4a1 is the main regulator of cardiac post-ischemic injury

To investigate the upstream regulators of post-ischemic cardiac injury, the GSE214122 dataset was analyzed. This dataset includes three mice in the sham surgery group and three in the myocardial infarction (MI) model group. Differential gene expression analysis was conducted using DESeq2, with a volcano plot visualizing the overall distribution of gene expression changes. A heatmap was generated to display the 20 most significantly differentially expressed genes. Among these, Nr4a1 exhibited significant differential expression, prompting further analysis of the raw expression values of the Nr4a1 family genes through an additional heatmap and a violin plot to compare expression levels between the sham surgery and MI model groups.

To complement this analysis, the GSE221539 dataset, comprising four mice in the sham operation group and four in the MI group, and the GSE236374 dataset, including three mice in the sham operation group and three in the 7-day post-MI model group, were also analyzed. Intersection genes from these two datasets were identified using R, and their expression data were extracted across the sham, MI, and 7-day MI groups. Differential expression analysis of these intersection genes was performed using DESeq2. Gene Ontology (GO) enrichment analysis revealed significant functional enrichment of differential genes in Biological Processes (BP), Molecular Functions (MF), and Cellular Components (CC). KEGG pathway analysis identified key signaling pathways implicated in the differential gene sets. BP enrichment analysis highlighted mitochondrial-related biological processes as predominant, prompting Gene Set Enrichment Analysis (GSEA) focused on mitophagy and mitochondrial fission pathways.

**Figure 1:**
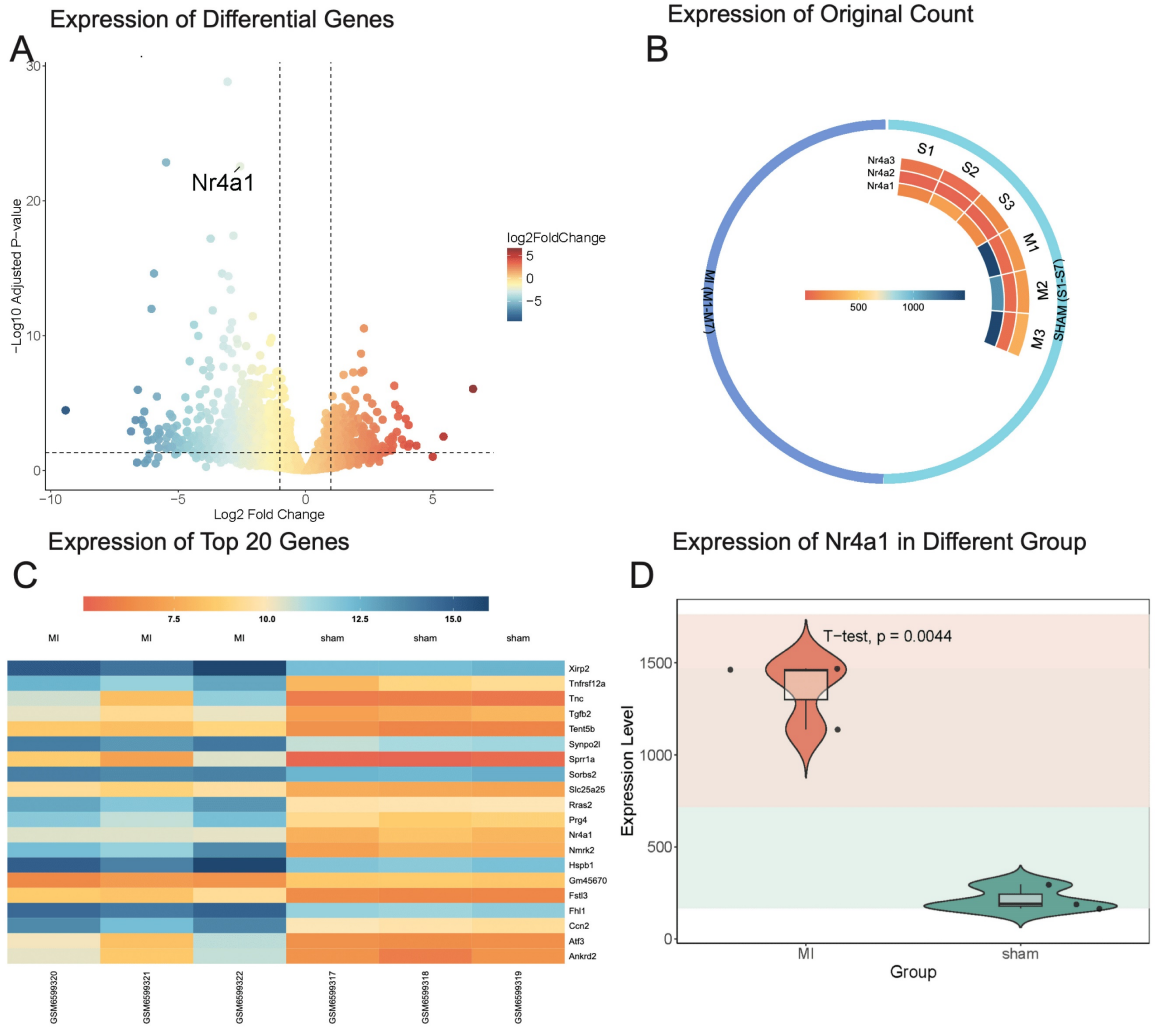
** Nr4a1 is the main regulator of cardiac post-ischemic injury. (A)** The x-axis represents the Log2 Fold Change, with red indicating high expression and blue indicating low expression. The y-axis represents the adjusted p-value, where higher values indicate smaller p-values and greater statistical significance. Nr4a1 is identified as one of the significantly upregulated genes.** (B)** Expression levels of Nr4a1 in the sham group are 166, 295, and 190, while in the MI model group, they are 1468, 1140, and 1460, respectively. For Nr4a2, expression levels in the sham group are 16, 22, and 23, compared to 63, 62, and 73 in the MI model group. Similarly, Nr4a3 expression levels in the sham group are 97, 64, and 175, whereas in the MI model group, they are 256, 265, and 359.** (C)** The heatmap uses a color scale to depict gene expression values following variance stabilizing transformation (VST), where darker colors correspond to higher expression levels and lighter colors indicate lower expression. Genes are sorted by adjusted p-value, with the MI group showing significant changes in the expression of multiple genes associated with myocardial remodeling, including Fhl1, Nmrk2, and Nr4a1, suggesting their potential involvement in post-myocardial infarction pathological processes.** (D)** Nr4a1 exhibits significantly higher expression in the MI model group compared to the sham group, with a p-value of 0.0044.

The differential gene expression analysis identified Nr4a1 as significantly differentially expressed (baseMean = 760.76, log2FoldChange = -2.57, adjusted p-value = 2.79 × 10⁻²³) (Figure 1A). Within the Nr4a1 family, Nr4a1 expression was lower in the sham surgery group and higher in the MI model group (Figure 1B). Among the top 20 most significantly differentially expressed genes, the top three were Fhl1, Nmrk2, and Nr4a1, with adjusted p-values of 1.48 × 10⁻²⁹, 1.41 × 10⁻²³, and 2.79 × 10⁻²³, respectively. Nr4a1 exhibited a statistically significant difference in expression between the sham and MI model groups (Figure 1D). These findings highlight the potential regulatory role of Nr4a1 in post-ischemic injury and the mitochondrial processes involved.

### Increased Nr4a1 exacerbates cardiac damage

We examined the involvement of a specific nuclear receptor in cardiac damage caused by interrupted blood flow. The receptor's expression was found to be significantly elevated in both damaged heart tissue and isolated cardiac endothelial cells, as evidenced by molecular and protein analyses. This upregulation was observed at both the transcriptional and protein levels, indicating a potential role in the pathological response to cardiac injury.

**Figure 2:**
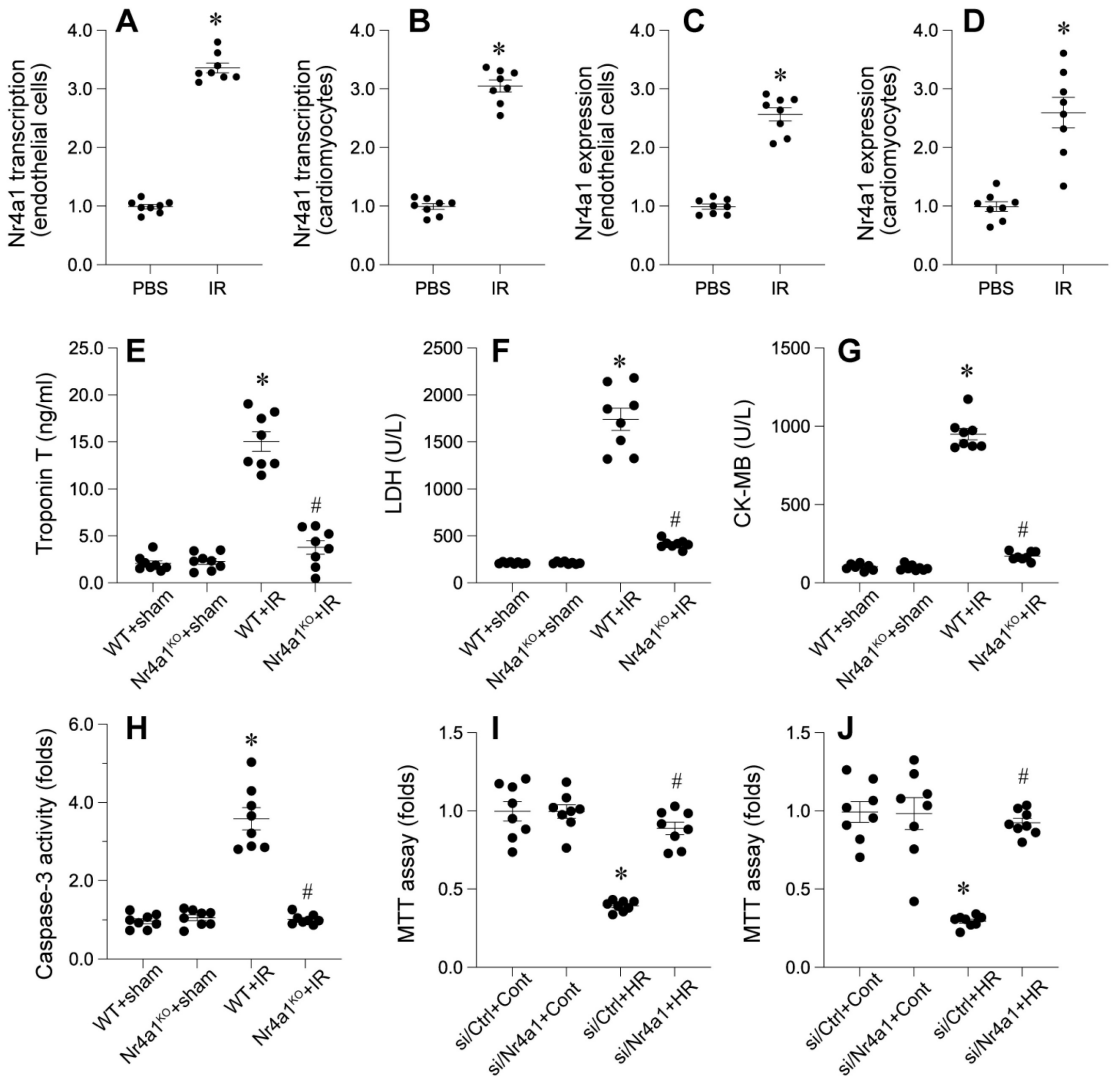
** Nr4a1 is upregulated during IR injury and promotes cardiac injury and cell death. (A-D)** Transcriptional and protein expression levels of Nr4a1 in cardiac microvascular endothelial cells (A, C) and cardiomyocytes (B, D) were significantly elevated after ischemia-reperfusion (IR) injury, as assessed by qPCR and Western blotting. **(E-G)** Serum levels of myocardial injury markers, including troponin T (E), lactate dehydrogenase (LDH, F), and creatine kinase-MB (CK-MB, G), were significantly increased in WT mice subjected to IR injury compared to Nr4a1-KO mice. **(H)** Caspase-3 activity, a marker of apoptosis, was elevated in WT+IR mice but was markedly reduced in Nr4a1-KO mice, indicating a protective effect of Nr4a1 deletion against apoptotic cell death. **(I-J)** MTT assays showing decreased cell viability in endothelial cells (I) and cardiomyocytes (J) exposed to HR injury, with Nr4a1 knockdown (si Nr4a1) restoring viability under HR conditions. *P < 0.05 vs. WT+Sham; #P < 0.05 vs. WT+IR.

*In vivo*, Nr4a1-knockout (Nr4a1-KO) mice exhibited significant attenuation of IR-induced cardiac injury markers, including CK-MB, troponin T, and LDH, compared to their wild-type (WT) counterparts (Figure 2E-G). We also observed a substantial reduction in apoptotic activity, as evidenced by decreased caspase-3 activation in Nr4a1-KO mice compared to WT mice (Figure 2H). Complementary *in vitro* findings indicated enhanced viability of cardiomyocytes (Figure 2I) and CMECs (Figure 2J) lacking Nr4a1 under HR conditions. Collectively, these data underscore the detrimental role of Nr4a1 in exacerbating cardiac damage during IR injury.

### Loss of Nr4a1 attenuates myocardial inflammation and restores endothelial barrier integrity

Inflammation is a key contributor to tissue damage caused by interrupted blood flow. Molecular analysis showed a marked increase in pro-inflammatory signals, including specific cytokines and chemokines, in response to cardiac injury. Notably, the absence of a specific nuclear receptor substantially mitigated these inflammatory reactions.

**Figure 3:**
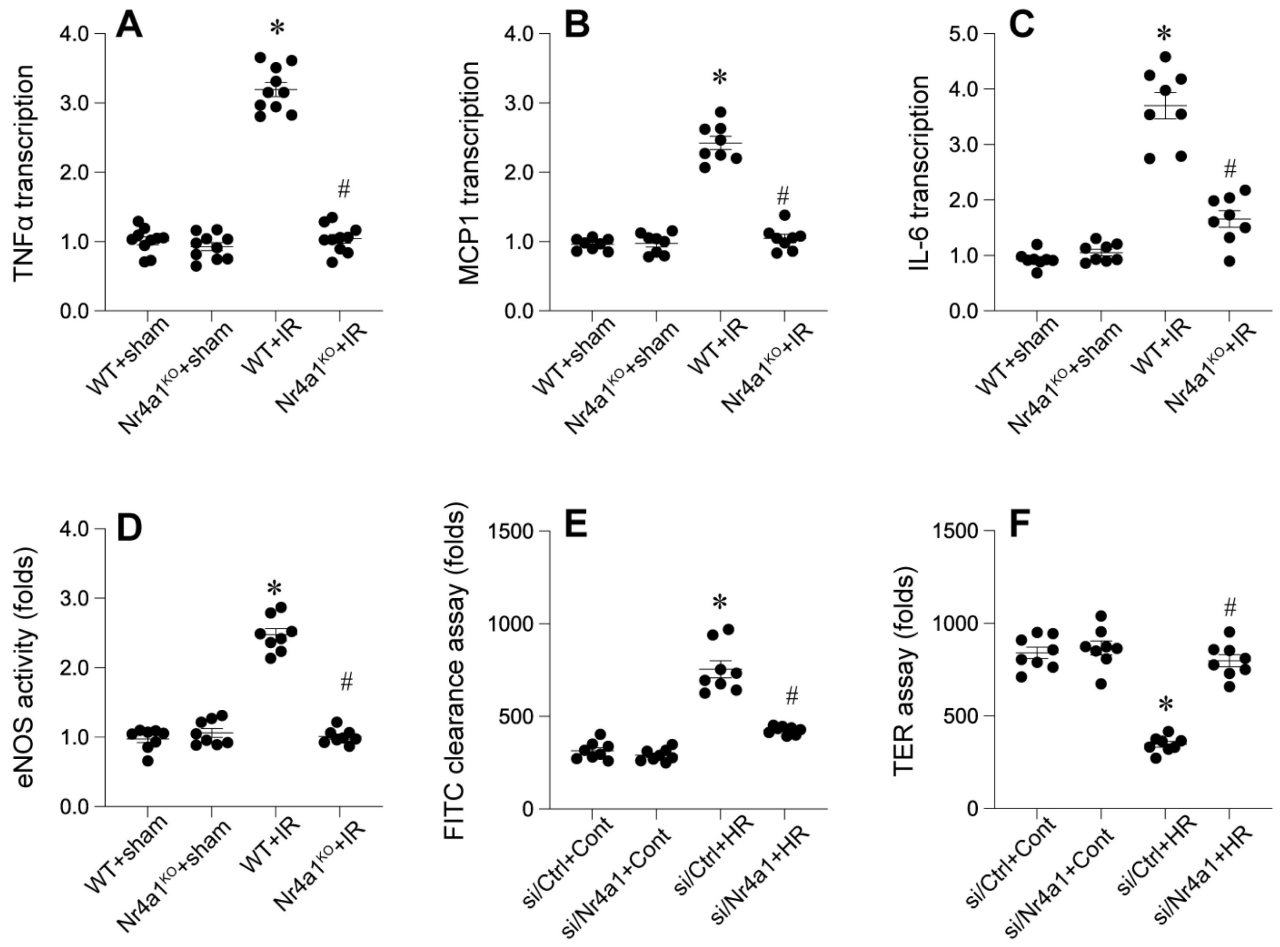
** Nr4a1 affecs myocardial inflammation response and endothelial function. (A-C)**. qPCRs were used to evaluate the transcription of inflammatory cytokines, including interleukin-6 (IL-6), tumor necrosis factor-alpha (TNF-α), and monocyte chemoattractant protein-1 (MCP-1). **(D)**. ELISA was used to analyze the changes of eNOS activity. **(E-F)**. FITC-dextran permeability assays and transendothelial electrical resistance (TER) measurements were used to observe the endothelial function. *P < 0.05 vs. WT+Sham; #P < 0.05 vs. WT+IR.

To further evaluate endothelial barrier function, we examined eNOS activity using ELISA. WT mice displayed disrupted eNOS activity, indicative of compromised endothelial integrity (Figure 3D). However, Nr4a1-KO mice retained high eNOS activity, reflecting preserved barrier functionality. FITC-dextran permeability assays and transendothelial electrical resistance (TER) measurements corroborated these findings (Figure 3E-F), with significantly lower permeability and higher TER values in Nr4a1-KO CMECs compared to WT controls. Together, these results suggest that Nr4a1 exacerbates inflammation and endothelial barrier dysfunction during IR injury.

### Nr4a1 suppresses mitochondrial function

**Figure 4:**
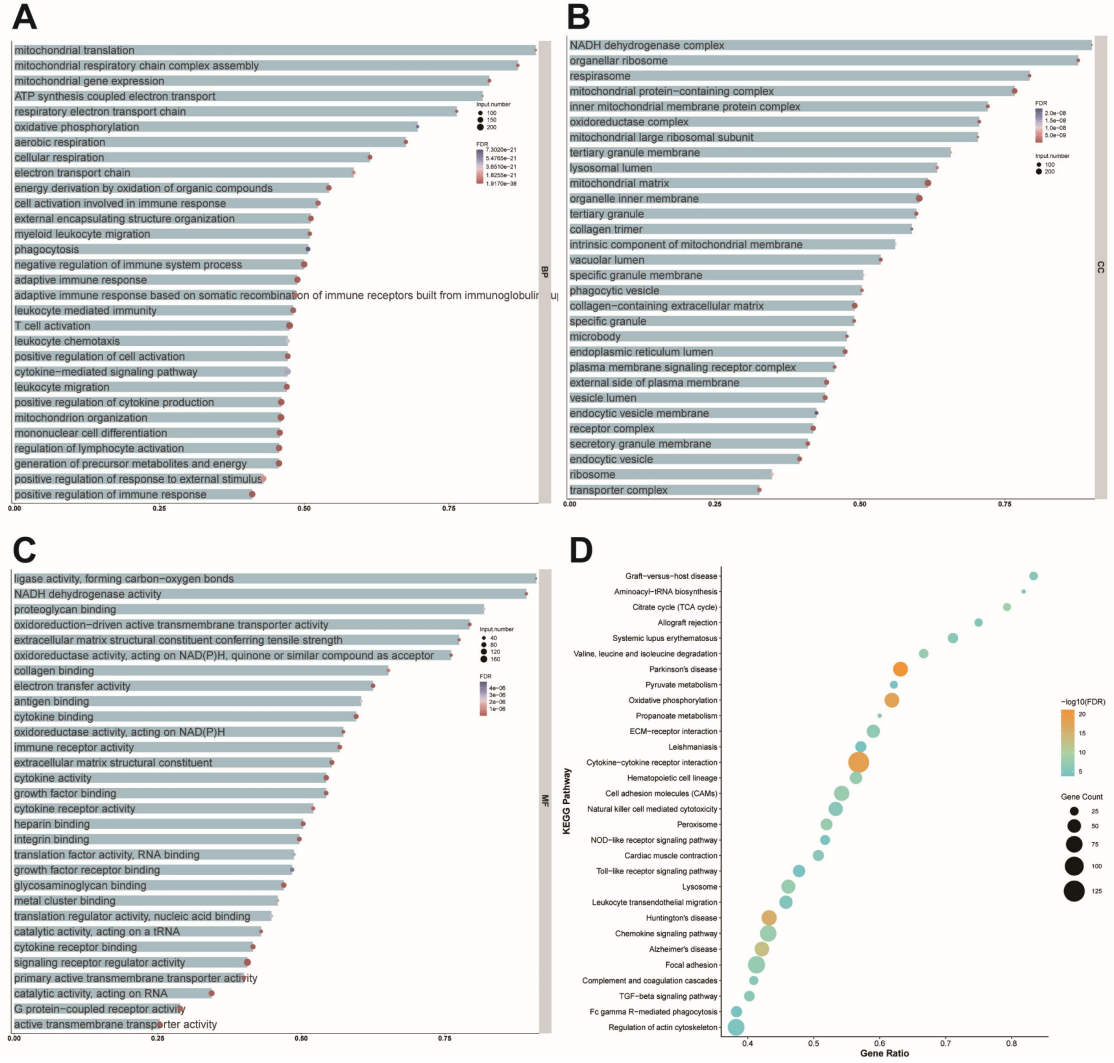
** Mitochondrial function is affected by cardiac post-ischemic injury. (A)** Bubble size represents the number of enriched genes, with larger bubbles indicating more enriched genes. Bubble color represents the FDR value, where redder colors indicate smaller FDR values and greater significance. The x-axis shows the proportion of enriched genes. Biological processes are primarily enriched in mitochondrial translation (63/70, 90%; FDR = 8.04 × 10⁻²²), mitochondrial respiratory chain complex assembly (73/84, 86.9%; FDR = 2.14 × 10⁻²⁶), and mitochondrial gene expression (82/100, 82%; FDR = 1.03 × 10⁻²⁶). **(B)** The size of each bubble represents the number of enriched genes, with bubble color indicating FDR values (redder colors correspond to smaller FDR values and higher significance). The x-axis shows the proportion of enriched genes. Cellular components are significantly enriched in the NADH dehydrogenase complex (36/40, 90%; FDR = 5.68 × 10⁻¹⁹), organellar ribosome (71/81, 87.65%; FDR = 6.60 × 10⁻²⁰), and respirasome (65/82, 79.27%; FDR = 4.19 × 10⁻²⁵). **(C)** Bubble size indicates the number of enriched genes, with bubble color denoting FDR values (redder colors indicate smaller FDR values and greater significance). The x-axis represents the proportion of enriched genes. Molecular functions are significantly enriched in ligase activity forming carbon-oxygen bonds (20/22, 90.91%; FDR = 4.89 × 10⁻⁶), NADH dehydrogenase activity (33/37, 89.19%; FDR = 1.71 × 10⁻¹⁵), and proteoglycan binding (27/33, 81.81%; FDR = 2.59 × 10⁻⁶). **(D)** The size and color of each bubble represent the number of enriched genes and the FDR value, respectively, with redder colors indicating greater significance. The x-axis shows the proportion of enriched genes. KEGG pathway analysis reveals significant enrichment in graft-versus-host disease (25/30, 83.33%; FDR = 2.17 × 10⁻⁷), aminoacyl-tRNA biosynthesis (18/22, 81.81%; FDR = 2.25 × 10⁻⁷), and the citrate cycle (23/29, 79.31%; FDR = 1.20 × 10⁻¹⁰).

To elucidate the molecular changes following cardiac injury, we analyzed two datasets from mouse models of myocardial infarction. The results provided valuable insights into the biological processes, cellular components, and molecular functions affected by cardiac injury. Notably, the analysis highlighted the critical role of mitochondrial-related processes, including protein synthesis, respiratory chain assembly, and gene expression. Mitochondrial translation involved 70 total genes, with 63 enriched genes accounting for 90% of the total (FDR = 8.04 × 10⁻²²), while mitochondrial respiratory chain complex assembly included 84 total genes, with 73 enriched genes (86.90%; FDR = 2.14 × 10⁻²⁶). Mitochondrial gene expression encompassed 100 total genes, with 82 enriched genes (82%; FDR = 1.03 × 10⁻²⁶). These findings underscore the prominence of mitochondrial processes in the dataset (Figure 4A). Cellular component (CC) analysis highlighted the NADH dehydrogenase complex, organellar ribosome, and respirasome. The NADH dehydrogenase complex involved 40 total genes, with 36 enriched genes (90%; FDR = 5.68 × 10⁻¹⁹), while the organellar ribosome comprised 81 total genes, with 71 enriched genes (87.65%; FDR = 6.60 × 10⁻²⁰). The respirasome included 82 total genes, with 65 enriched genes (79.27%; FDR = 4.19 × 10⁻²⁵), emphasizing their role in mitochondrial function (Figure 4B). Molecular function (MF) analysis further reinforced the importance of mitochondrial processes, with significant enrichment observed in ligase activity forming carbon-oxygen bonds, NADH dehydrogenase activity, and proteoglycan binding.

Ligase activity involved 22 total genes, of which 20 were enriched (90.91%; FDR = 4.89 × 10⁻⁶), NADH dehydrogenase activity included 37 total genes, with 33 enriched genes (89.19%; FDR = 1.71 × 10⁻¹⁵), and proteoglycan binding encompassed 33 total genes, with 27 enriched genes (81.81%; FDR = 2.59 × 10⁻⁶) (Figure 4C). KEGG pathway analysis identified graft-versus-host disease, aminoacyl-tRNA biosynthesis, and the citrate cycle (TCA cycle) as significantly enriched pathways. Graft-versus-host disease involved 30 total genes, with 25 enriched genes (83.33%; FDR = 2.17 × 10⁻⁷), aminoacyl-tRNA biosynthesis included 22 total genes, with 18 enriched genes (81.81%; FDR = 2.25 × 10⁻⁷), and the citrate cycle comprised 29 total genes, with 23 enriched genes (79.31%; FDR = 1.20 × 10⁻¹⁰), reflecting the critical role of these pathways in mitochondrial energy metabolism (Figure 4D). These findings collectively emphasize the pivotal roles of mitochondrial processes, structures, and pathways in cellular function, providing valuable insights for understanding the molecular mechanisms underlying mitochondrial regulation.

### Fis1-related mitochondrial fission is affected during cardiac post-ischemic injury

**Figure 5:**
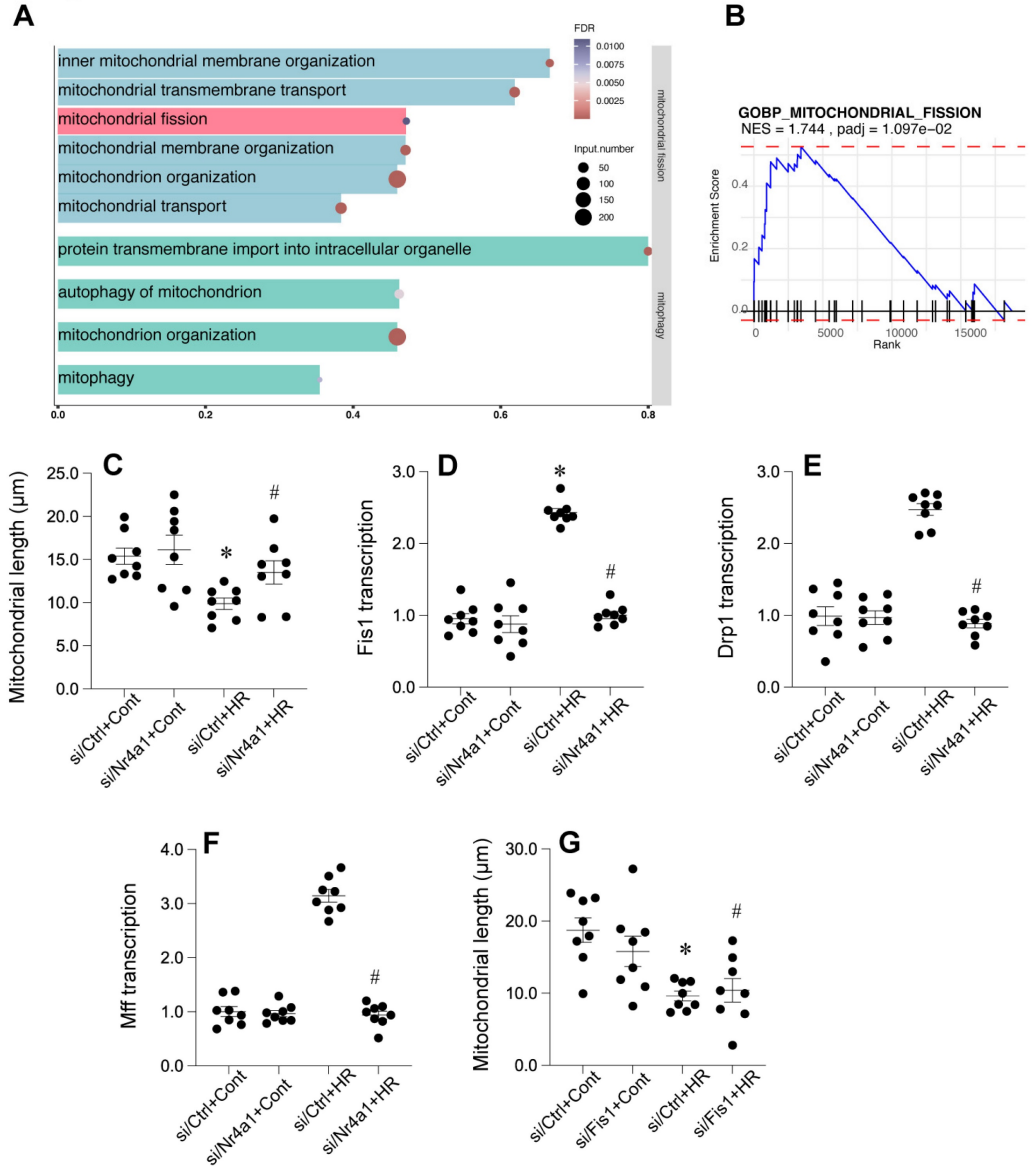
** Nr4a1 regulates mitochondrial fission by modulating key fission-related factors. (A)** Gene Ontology (GO) enrichment analysis highlighting pathways associated with mitochondrial fission and membrane organization under hypoxia-reoxygenation (HR) conditions. **(B)** Gene Set Enrichment Analysis (GSEA) of the mitochondrial fission pathway demonstrating its significant activation under HR stress (NES = 1.744, P-adjusted = 1.097e-02). **(C)** Quantification of mitochondrial length showing HR-induced mitochondrial fragmentation, which is mitigated by Nr4a1 knockdown (siNr4a1+HR). **(D-E)** Transcriptional analysis of Fis1 and Drp1, two key regulators of mitochondrial fission, showing HR-induced upregulation that is suppressed by Nr4a1 knockdown. **(F)** Mff transcription levels showing similar HR-induced changes that are inhibited by NR4A1 knockdown. **(G)** Reactivation of Fis1 in si Nr4a1-treated cells restores mitochondrial fragmentation, indicating Fis1's central role in Nr4a1-mediated mitochondrial dysfunction. #P<0.05 vs. siCtrl+HR; *P<0.05 vs. siCtrl+Cont.

To further illuminate the potential mechanism undelrying mitochodnrial dysfunciton, we further analyzed the GSE221539 dataset and the GSE236374 dataset. Our results demonstrated that GSEA revealed significant enrichment in multiple mitochondrial-related pathways, including inner mitochondrial membrane organization, mitochondrial transmembrane transport, and mitochondrial fission (Figure 5A), with mitochondrial fission pathway showing a normalized enrichment score (NES) of 1.744 and adjusted p-value of 0.0197 (Figure 5B). Valdation studies showed that mitochondrial length was reduced by HR injury and returned to normal levels once deleiton of Nr4a1 (Figure 5C). Analysis of key mitochondrial fission-related genes showed that Fis1 transcription was significantly upregulated in by HR injury (Figure 5D) but suppressed by Nr4a1 deletion, with Drp1 and Mff expression patterns following similar trends (Figures 5E and 5F). Additional mitochondrial length measurements confirmed that loss of Fis1 could reverse HR-induced changes in mitochondrial length (Figure 5G), collectively suggesting that HR injury may influence Fis1-related mitochondrial fission.

### Nr4a1 knockdown protects mitochondrial integrity through Fis1-related mitochondrial fission

**Figure 6:**
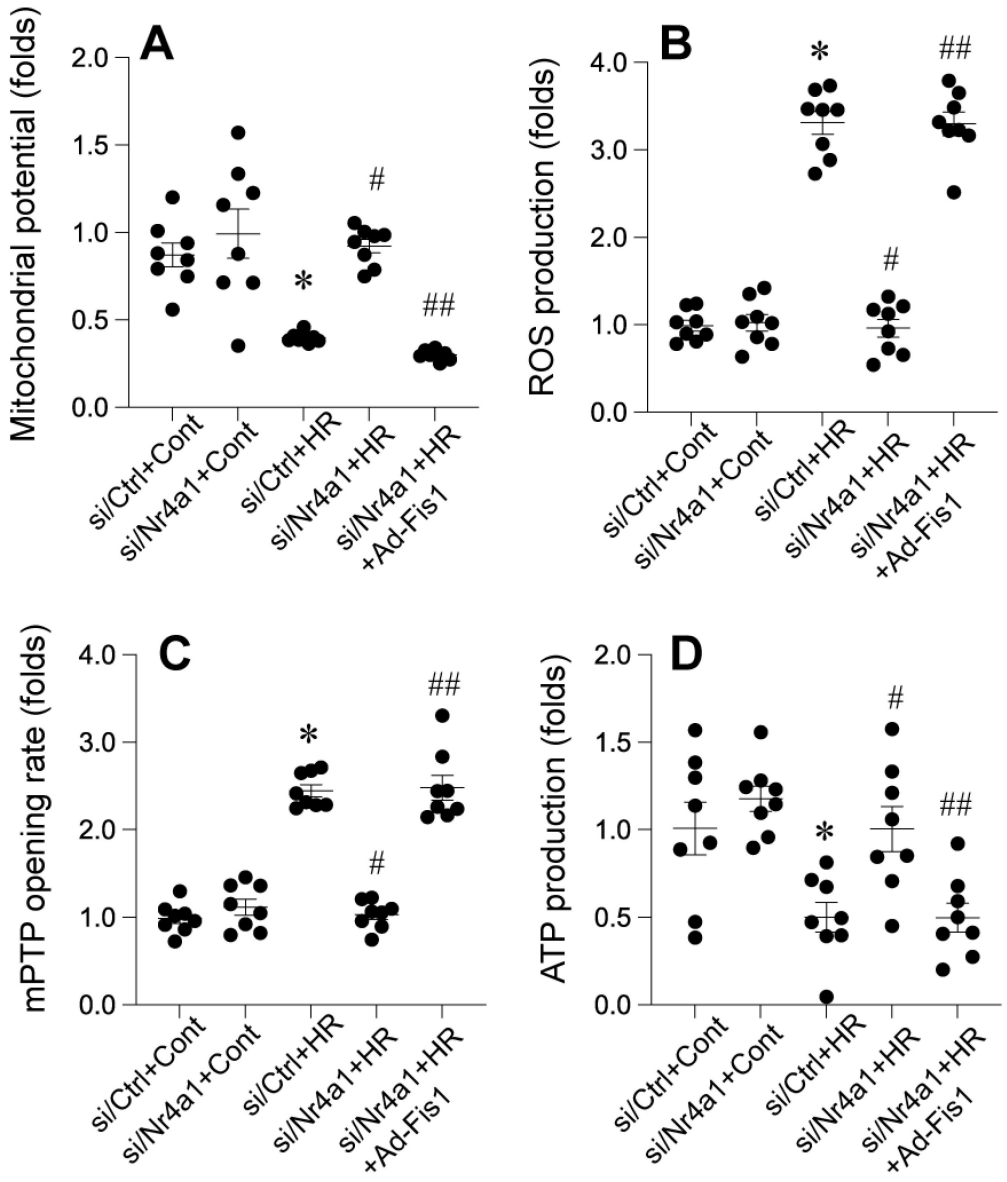
** Nr4a1 knockdown preserves mitochondrial integrity and reduces oxidative stress via modulation of mitochondrial fission. (A)** Measurement of mitochondrial membrane potential (ΔΨm), showing that Nr4a1 knockdown restores ΔΨm under HR conditions, while Fis1 reactivation reverses this effect. **(B)** Reactive oxygen species (ROS) production significantly increases under HR conditions but is reduced by Nr4a1 knockdown, with Fis1 reactivation reinstating oxidative stress. **(C)** Analysis of mitochondrial permeability transition pore (mPTP) opening rates, showing protective effects of Nr4a1 knockdown against HR-induced mitochondrial damage. **(D)** ATP production levels, demonstrating that Nr4a1 knockdown restores bioenergetic function under HR stress, while Fis1 reactivation disrupts ATP production. These findings highlight the central role of Nr4a1 in regulating mitochondrial function and its downstream effects via Fis1. #P<0.05 vs. siCtrl+HR; *P<0.05 vs. siCtrl+Cont; ##P<0.05 vs. siNr4a1+HR.

Nr4a1 knockdown confers significant protection against HR-induced mitochondrial dysfunction, oxidative stress, and bioenergetic failure, while Fis1 reactivation abolishes these protective effects. Under HR conditions, mitochondrial membrane potential (ΔΨm) was markedly reduced (Figure 6A), indicating impaired mitochondrial integrity, which was substantially restored in Nr4a1-silenced cells. However, reactivation of Fis1 reversed this restoration, leading to a significant decline in ΔΨm (Figure 6A). In parallel, ROS production was significantly elevated in HR-treated control cells, reflecting oxidative stress. This increase was mitigated by Nr4a1 knockdown, but Fis1 reactivation reinstated high ROS levels, underscoring the role of Fis1 in driving mitochondrial oxidative damage (Figure 6B). Furthermore, HR-induced mitochondrial permeability transition pore (mPTP) opening, a hallmark of mitochondrial dysfunction, was significantly suppressed in Nr4a1-deleted cells, while Fis1 reactivation restored mPTP opening to levels observed in HR-treated controls (Figure 6C). ATP production, which was drastically reduced under HR conditions, was significantly improved following Nr4a1 knockdown, indicating enhanced mitochondrial bioenergetics. However, Fis1 reactivation negated this improvement, demonstrating its detrimental role in energy metabolism under stress (Figure 6D). Collectively, these findings highlight the critical role of Nr4a1 in regulating mitochondrial function during HR injury and establish Fis1 as a key downstream mediator of mitochondrial dysfunction and energy failure, providing mechanistic insight into the interplay between Nr4a1 and Fis1 in mitochondrial homeostasis during ischemia-reperfusion injury.

### Nr4a1 inhibits Parkin-dependent mitophagy

**Figure 7:**
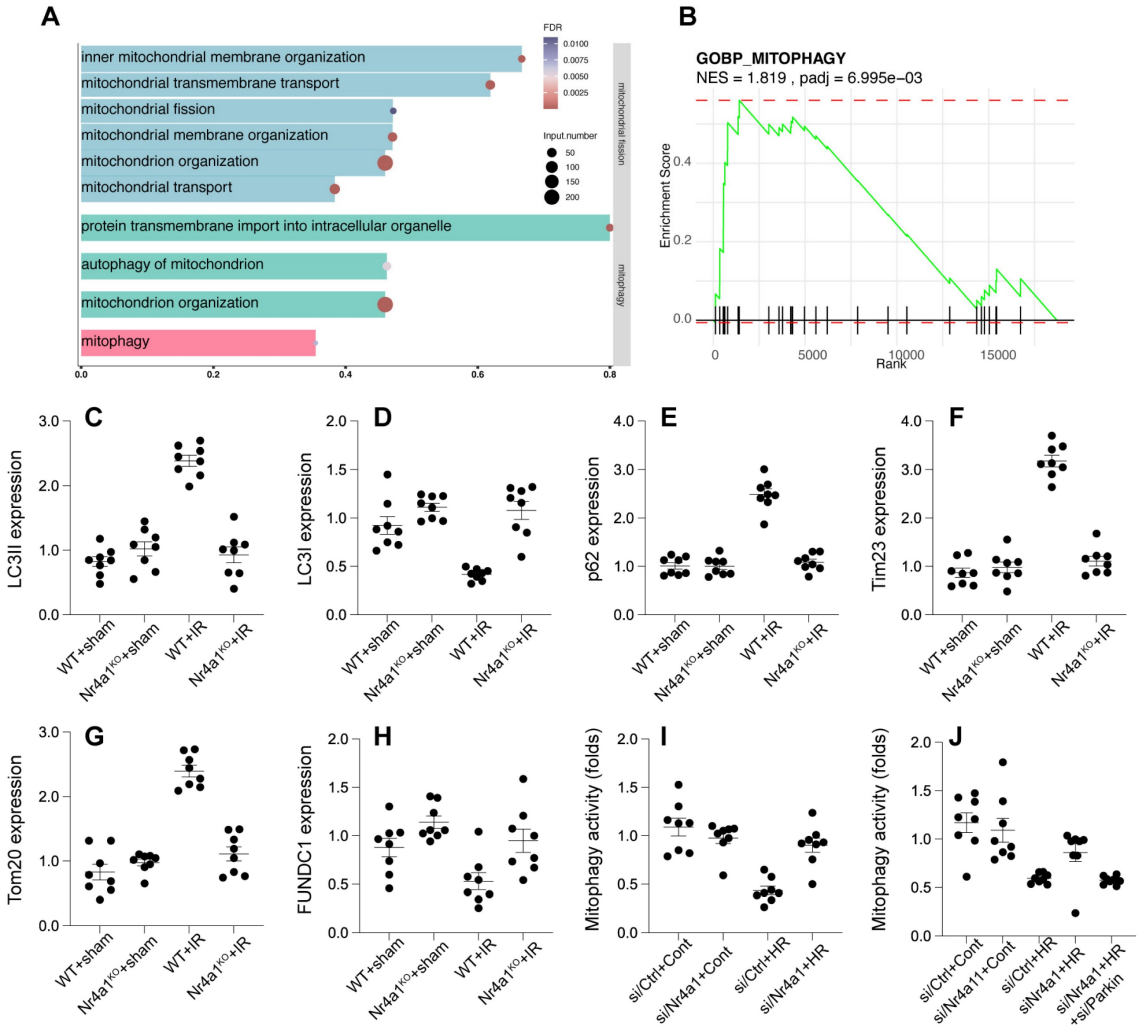
** Nr4a1 regulates mitophagy through Parkin-mediated pathways under HR stress. (A)** GO enrichment analysis showing activation of pathways related to mitophagy and mitochondrial quality control. **(B)** GSEA of the mitophagy pathway, indicating its suppression under HR conditions (NES = 1.819, P-adjusted = 6.995e-03). **(C-D)** LC3-II/I ratios indicating enhanced autophagic flux in Nr4a1-knockout mice under HR conditions. **(E-F)** Expression of p62 and Trim23, showing the impaired mitophagic flux under HR conditions that is rescued in Nr4a1-knockout mice. **(G-H)** Parkin and Tom20 expression, indicating Nr4a1's role in mitophagy regulation. **(I-J)** Mitophagy activity analysis showing that Nr4a1 knockdown enhances mitophagic flux, while Parkin silencing negates these effects. These results underscore the critical role of Nr4a1 in suppressing mitophagy during HR injury. #P<0.05 vs. WT+IR; *P<0.05 vs. WT+sham.

To figure out whether other mitochondria-related molecular mechanism underlying post-ischemic injury, we further analyzed the GSE221539 dataset and the GSE236374 dataset. Enrichment analysis highlighted the involvement of mitophagy in the regulation of mitochondrial function. In the biological process of mitophagy, a total of 80 genes were identified, with 37 enriched genes, accounting for 46.25% of the total genes (FDR = 0.0053). The size of the bubbles in the visualization indicates the number of enriched genes, while the color reflects the FDR value, with redder bubbles representing greater statistical significance (Figure 7A). Gene Set Enrichment Analysis (GSEA) further demonstrated that mitophagy was significantly enriched in upregulated genes, with a Normalized Enrichment Score (NES) of 1.81 and an adjusted p-value (padj) of 6.99 × 10⁻³ (Figure 7B). These findings suggest that mitophagy plays a pivotal role in mitochondrial regulatory processes under the studied conditions. Western blot analysis revealed that HR injury reduced LC3-II/I ratios and Tim23/Tom20 expression, an effect that was followed by p62 accumulation (Figure 7C-H), indicating impaired mitophagic flux (Figure 7I). However, these deficits were reversed in Nr4a1-KO cells. Deletion of Parkin through adenovirus transfection in Nr4a1-KO cells abolished these protective effects (Figure 7C-J), underscoring the critical role of Parkin in facilitating mitophagy under NR4A1-deficient conditions. These results suggest that Nr4a1 impedes Parkin-mediated mitophagy, thereby contributing to mitochondrial dysfunction during IR injury.

### Parkin-mediated mitophagy counteracts pathological mitochondrial fission

**Figure 8:**
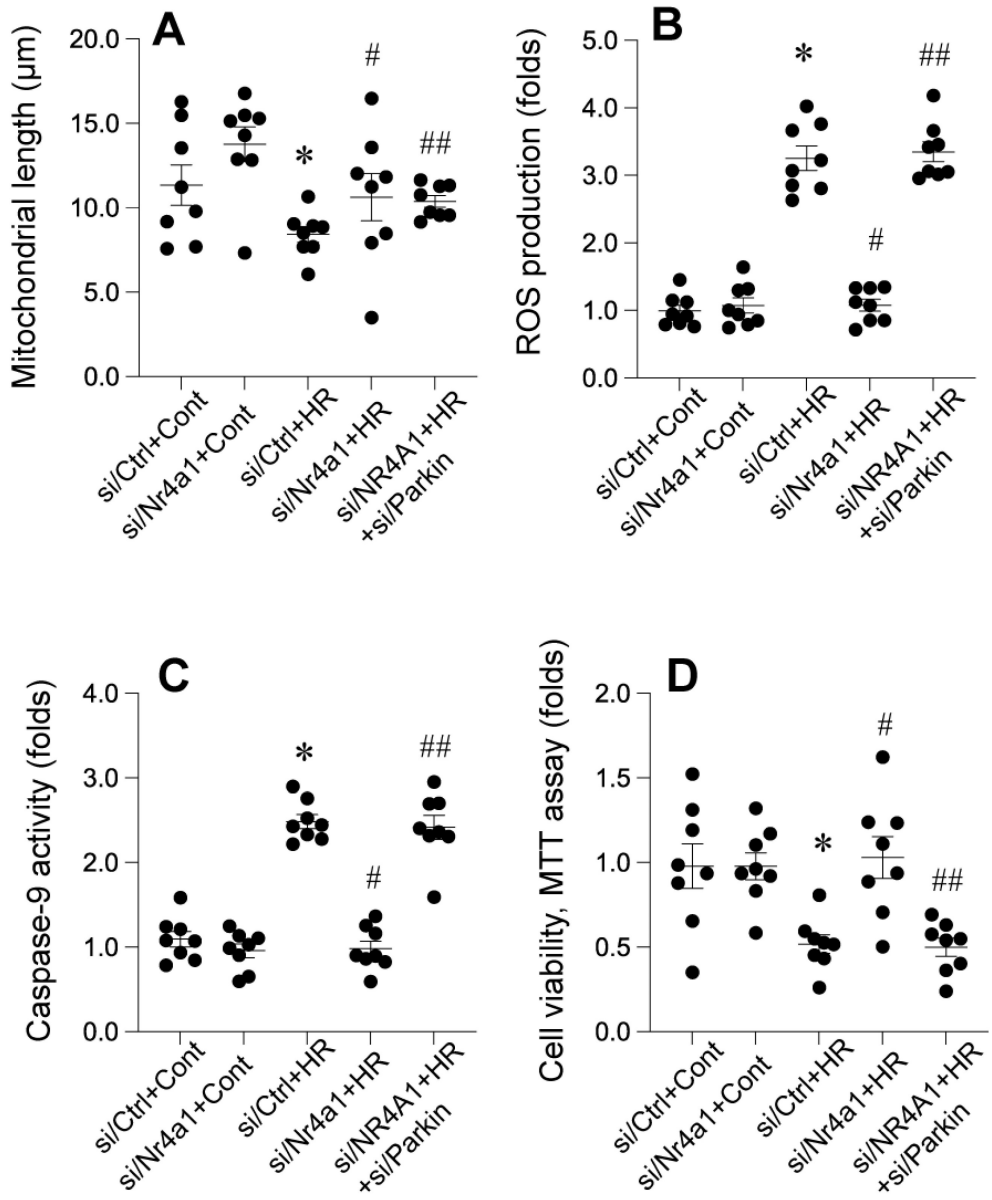
** Parkin-dependent mitophagy is essential for Nr4a1-mediated mitochondrial protection under HR conditions. (A)** Measurement of mitochondrial length, showing that Nr4a1 knockdown restores mitochondrial integrity under HR conditions, an effect abolished by Parkin silencing. **(B)** ROS production levels, indicating that Parkin-mediated mitophagy reduces oxidative stress under Nr4a1 knockdown, while Parkin silencing reinstates elevated ROS. **(C)** Caspase-9 activity, demonstrating that Parkin-dependent mitophagy inhibits apoptosis in Nr4a1-deficient cells under HR stress. **(D)** Cell viability assessed via MTT assay, showing that Nr4a1 knockdown enhances cellular resilience, while Parkin silencing reduces viability under HR conditions. These results establish Parkin as a critical mediator of Nr4a1-driven mitochondrial protection. #P<0.05 vs. siCtrl+HR; *P<0.05 vs. siCtrl+Cont; ##P<0.05 vs. siNr4a1+HR.

Nr4a1 knockdown significantly attenuates HR-induced mitochondrial fragmentation, oxidative stress, apoptosis, and loss of cellular viability, with these protective effects being dependent on FUNDC1-mediated mitophagy. Figure 8 A demonstrates that HR exposure significantly reduced mitochondrial length, indicating excessive mitochondrial fragmentation. This effect was reversed in Nr4a1-deficient cells, where mitochondrial length was restored, suggesting the suppression of pathological mitochondrial fission. However, silencing Parkin in Nr4a1-deficient cells eliminated this restoration, resulting in fragmented mitochondria, indicating the essential role of Parkin in maintaining mitochondrial morphology. Figure 8B shows that HR significantly increased ROS production, a hallmark of oxidative stress. Nr4a1 knockdown markedly reduced ROS levels, indicating reduced oxidative damage, but Parkin silencing reinstated elevated ROS production, suggesting that Parkin-dependent mitophagy is critical for limiting oxidative stress. Similarly, Figure 8C highlights the effect of Nr4a1 knockdown on apoptosis. HR treatment led to significantly increased caspase-9 activity, indicative of mitochondrial apoptotic signaling. Nr4a1 deficiency suppressed this activity, while Parkin silencing in Nr4a1-deficient cells restored caspase-9 activity to levels observed in HR-treated controls, further demonstrating the importance of Parkin in inhibiting apoptosis. Finally, Figure 8D illustrates cell viability as measured by the MTT assay. HR significantly reduced cellular viability, but Nr4a1 knockdown rescued this loss, reflecting enhanced cell survival. However, silencing Parkin in Nr4a1-deficient cells negated this protective effect, leading to a substantial decline in viability. Collectively, these findings demonstrate that Nr4a1 knockdown protects against HR-induced injury by maintaining mitochondrial integrity, reducing oxidative stress and apoptosis, and enhancing cell survival, with these effects being critically dependent on Parkin-mediated mitophagy.

## Discussion

This study reveals the involvement of a specific nuclear receptor in cardiac damage caused by interrupted blood flow, highlighting its impact on mitochondrial function. We show that this receptor is significantly upregulated in response to cardiac injury and worsens cardiac damage by disrupting mitochondrial balance. The receptor promotes mitochondrial fragmentation and inhibits a key quality control process, thereby contributing to mitochondrial dysfunction. These findings suggest potential therapeutic targets for reducing cardiac damage.

Mitochondrial quality control relies on the balance between mitochondrial division and a specific cellular recycling process, especially during stress. However, excessive division can produce defective mitochondrial fragments, impairing energy production and inducing cell death 33. In contrast, the cellular recycling process plays a vital role in removing damaged mitochondria, maintaining cellular balance. Our data reveal that Nr4a1 orchestrates a pathological shift in mitochondrial dynamics by enhancing fission and inhibiting mitophagy. Specifically, Nr4a1 upregulates Fis1 expression, facilitating Drp1 recruitment and mitochondrial fragmentation, while simultaneously downregulating Parkin, impairing mitophagic flux 34. These findings align with previous studies that have highlighted the detrimental effects of unchecked mitochondrial fission and suppressed mitophagy in various cardiac pathologies. The observed interplay between Nr4a1, Fis1, and Parkin underscores the complexity of mitochondrial regulation during IR injury and provides a mechanistic basis for the exacerbated cardiac damage observed in WT mice compared to Nr4a1-deficient counterparts.

Our results show that Nr4a1 contributes to endothelial barrier dysfunction and heightened permeability following IR injury. The disruption of VE-cadherin expression and increased FITC-dextran permeability in WT CMECs reflect a compromised endothelial barrier. In contrast, Nr4a1 deletion preserved VE-cadherin integrity, reduced permeability, and enhanced transendothelial electrical resistance (TER), highlighting the protective effects of Nr4a1 inhibition 35.

Furthermore, Nr4a1 was found to exacerbate inflammation during IR injury, as evidenced by the upregulation of pro-inflammatory cytokines such as IL-6, TNF-α, and MCP-1. These inflammatory mediators amplify endothelial damage and microvascular dysfunction, thereby contributing to the progression of IR injury 36. Our findings suggest that targeting Nr4a1 may not only alleviate mitochondrial dysfunction but also mitigate endothelial inflammation, offering dual protective effects in myocardial IR injury 37.

One of the most significant findings of this study is the reciprocal relationship between mitochondrial fission and mitophagy. While Fis1-mediated fission is necessary for mitochondrial turnover, excessive fission in the absence of compensatory mitophagy leads to mitochondrial accumulation and cellular apoptosis. Parkin-dependent mitophagy was shown to counteract pathological fission, promoting mitochondrial quality control and cell survival. This balance is disrupted by Nr4a1, which enhances Fis1 activity while inhibiting Parkin expression.

Our data reveal that restoring Parkin-mediated mitophagy in Nr4a1-deficient cells attenuates mitochondrial fragmentation, reduces ROS production, and stabilizes ΔΨm. Conversely, silencing Parkin negates the protective effects of Nr4a1 deletion, highlighting the critical role of mitophagy in mitigating fission-induced mitochondrial dysfunction 38. These findings emphasize the therapeutic potential of strategies aimed at modulating the fission-mitophagy axis in IR injury.

Our findings highlight Nr4a1 as a key regulator of mitochondrial dynamics and a potential therapeutic target for myocardial IR injury 39. By modulating the fission-mitophagy axis, Nr4a1 inhibition can alleviate mitochondrial dysfunction, reduce apoptosis, and preserve endothelial integrity. Future studies should explore the therapeutic potential of small-molecule inhibitors targeting Nr4a1 in preclinical models of IR injury. Investigating the role of other mitochondrial regulatory pathways and their interplay with Nr4a1 will further enhance our understanding of the mechanisms underlying IR-induced mitochondrial dysfunction. Additionally, the translation of these findings to human models will be crucial for developing effective therapies for ischemic heart disease.

This study provides novel insights into the mechanisms underlying mitochondrial regulation in cardiac microvascular endothelial cells during IR injury, highlighting the critical role of Nr4a1 and Parkin-mediated mitophagy. While previous studies have largely focused on cardiomyocytes, our findings emphasize the microvascular compartment as a pivotal mediator of IR injury, offering a deeper understanding of the pathological processes occurring at the microvascular level. The cardiac microvasculature is essential for maintaining adequate myocardial perfusion and oxygen delivery, and its dysfunction contributes to tissue injury, inflammation, and ultimately the progression of myocardial ischemic damage 40. By identifying Nr4a1 as a regulator of mitochondrial fission and Parkin-mediated mitophagy in CMECs, our study underscores the importance of mitochondrial homeostasis in preserving heart function during IR injury 41.

Our results demonstrate that Nr4a1 knockdown reduces mitochondrial fragmentation, oxidative stress, and endothelial apoptosis while preserving mitochondrial membrane potential and ATP production in CMECs under HR conditions. Notably, these protective effects were dependent on Parkin-mediated mitophagy, as silencing Parkin in Nr4a1-deficient cells abolished the improvements in mitochondrial integrity, oxidative stress, and cellular survival. These findings suggest that the microvascular endothelium relies on mitophagy as a critical defense mechanism to counteract mitochondrial damage and maintain cellular function during IR stress. Moreover, our study identifies the pathological role of Nr4a1 in exacerbating microvascular dysfunction through the promotion of Fis1-dependent mitochondrial fission and suppression of Parkin-mediated mitophagy, providing mechanistic insights into the interplay between these processes 42.

The novelty of our research lies in shifting the focus from cardiomyocyte-centric studies to the often-overlooked microvascular endothelium, which is highly susceptible to mitochondrial dysfunction during IR injury. CMECs are uniquely sensitive to fluctuations in mitochondrial dynamics, given their reliance on energy production and redox balance to maintain barrier integrity and vascular homeostasis. By demonstrating the detrimental effects of Nr4a1 activation in CMECs, we highlight a previously unrecognized pathway contributing to microvascular injury. Furthermore, the identification of Parkin as a critical modulator of mitophagy in CMECs establishes a novel therapeutic target for protecting the microvasculature during IR injury.

In summary, our study provides a significant advance in understanding the molecular mechanisms driving microvascular dysfunction during IR injury, emphasizing the role of mitochondrial dynamics in this process. Targeting the Nr4a1/mitophagy axis represents a promising therapeutic strategy to protect the cardiac microvasculature, thereby improving overall myocardial outcomes. These findings not only extend our knowledge of endothelial-specific mitochondrial regulation but also highlight the broader significance of maintaining microvascular health in the context of ischemic heart disease.

## Funding

This study is supported by the National Natural Science Foundation of China (No. 82200483, No. 82271806), the Natural Science Foundation of Guangdong Province (No. 2023A1515011687, No. 2022A1515110560), Guangzhou Science and Technology Plan Project (No. 2023A03J0697), and the Sun Yat-sen Memorial Hospital, Sun Yat-sen University's Scientific Research Sailing Project (No. YXQH2022017).
